# Highly Cellular Leiomyoma Mixed With a Focus of Adenomyosis

**DOI:** 10.7759/cureus.28129

**Published:** 2022-08-18

**Authors:** Henry J Nava

**Affiliations:** 1 Obstetrics and Gynecology, University of the Andes, Merida, VEN

**Keywords:** high cellularity, leiomyosarcoma, adenomyosis, leiomyoma, fibroids

## Abstract

The purpose of this case presentation was to highlight the importance of an adequate evaluation of images when suspicious of atypical leiomyoma and the importance of performing an extemporaneous biopsy during surgery to ensure the lesion is a benign muscular cell tumor.

Here, we present a case of a 34-year-old nulliparous woman who presented with a history of infertility and irregular menstrual cycles. A highly vascularized pelvic mass was visualized by Doppler ultrasound and a contrast MRI suggestive of uterine fibroid. Intraoperatively, the lesion was found adjacent to the uterus, with a second lesion deeper into the myometrium. The pathology reported a highly cellular leiomyoma with a focus of adenomyosis. Both lesions were extirpated without complications. The patient recuperated favorably within three months of follow-up. This case shows an example of a variety of the typical histology that can be found in uterine fibroids. Although the management of atypical leiomyomas could vary in different scenarios, conservative treatment is recommended if fertility wishes are present. In all cases, it is mandatory to exclude any possibilities of malignancy, like sarcoma, which would completely change the intraoperative management.

## Introduction

Uterine fibroid is a frequent benign tumor condition of the uterus, which is found in 70-80% of women above 50 years of age [1]. The causes and pathogenetic mechanisms of the development of leiomyomas are not fully understood. Still, the identification of progenitor stem cells (SCs) giving rise to maternal fibroid nodes and junction zones provides new evidence consistent with two molecular mechanisms - one of them is related to the MED12 gene mutations and proposes its onset in the subpopulation of embryonic myoblasts of the female reproductive system, which entails the formation of small fibroids and the second induced by predominantly epigenetic disturbances in limbal mesenchymal stromal cells (LM SC) caused by increased expression of the HMGA2 gene caused by hypomethylation and epigenetic deregulation, which is exacerbated by hypoxia and presented as single and usually large fibroids [2,3].

On the other hand, leiomyosarcomas are rare malignant uterine tumors associated with a significant proportion of uterine cancer deaths and can be suspected when performing imaging studies [4]. Changes in the lymph ganglion sizes and different visualization of the myometrium compared with endometrium allow a presumption of malignant sarcoma instead of leiomyomas with unusual patterns [5]. The relevance of imaging in the assessment of the genital tract is fundamental, especially in detecting uterine lesions [6]. It is well-known that non-degenerated fibroids can be regularly recognized while performing ultrasound or MRI, but there are many other similar histological findings like degenerated fibroids, fibroid variants, and fibroids with unusual growth patterns that can become a challenge, especially during the moment of selecting the most appropriate surgical treatment [5].

Cellular leiomyoma (CL) is a rare variant of uterine leiomyoma, which is difficult to detect clinically or even with imaging. However, immunohistochemical and molecular changes have been proved present during this variation [7]. Dundr et al. found immunohistochemical results of cellular leiomyomas with high expression of smooth muscle markers (calponin, desmin, smooth muscle actin, caldesmon, transgelin, smooth muscle myosin heavy chain, and smoothelin) commonly associated with co-expression of endometrial stromal markers like interferon-induced transmembrane protein 1 (IFITM1) and cluster of differentiation (CD)10 [7].

In women without fertility wishes and significant symptomatology, a hysterectomy for leiomyomas could be done. More conservative treatments like myomectomy are often preferred in premenopausal women. However, it is mandatory to rule out the possibility of malignancy before attempting medical or local excision management [3].

Uterine leiomyoma and adenomyosis can be present in the same uterus in about 15-57% of females with symptomatology, and in those cases, women can complain of more symptoms than when separate diagnostics are reported [8]. Adenomyosis is an endometrial tissue abnormally located in the uterine muscle, which is often present with other gynecological pathologies, such as leiomyomas. Still, there is no consensus on the exact histopathological and imaging perspectives, and the diagnosis criteria can vary between health care providers [6,8].

## Case presentation

**Figure 1:**
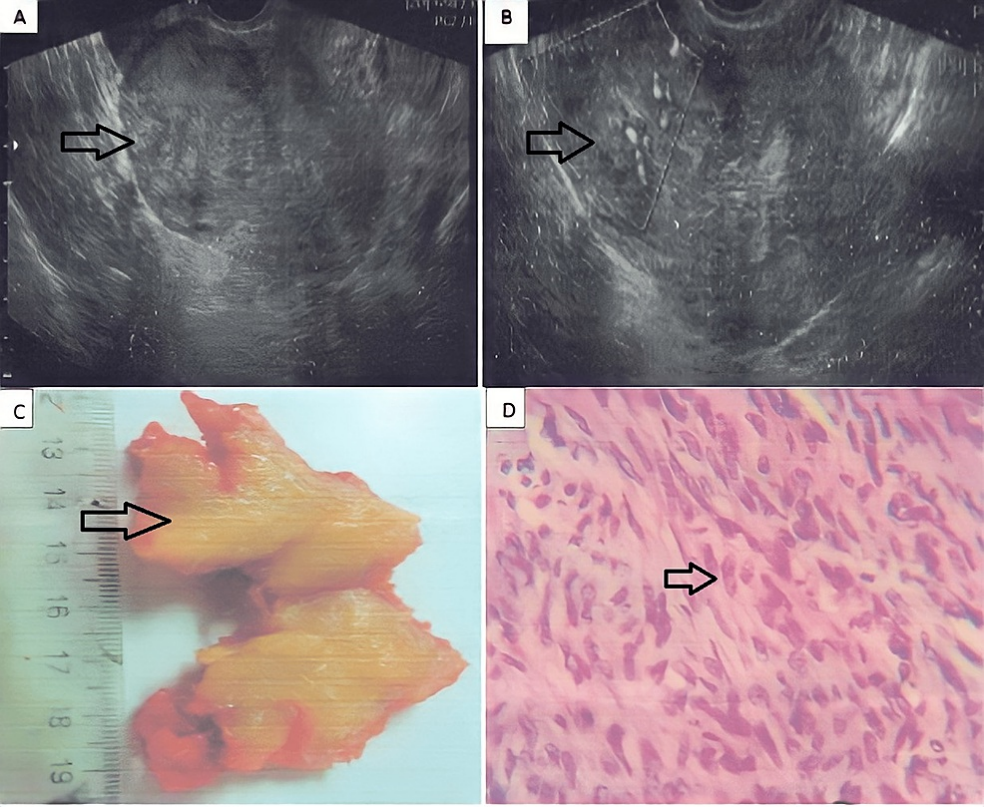
Ultrasound, macroscopic, and histological images of atypical leiomyoma. (A) Pelvic ultrasonography documented a homogeneous mass lesion right para uterine. (B) Previous image with doppler visualization. (C) Macroscopic lesion with irregular borders and thickness of the myometrium suggestive of leiomyoma. (D) Microscopic image of high cellular tumor leiomyoma composed of sheets of cells increased in number per unit area arranged in clusters of hyperchromatic nuclei with coarse chromatin.

A 34-year-old nulliparous woman presented with a history of primary infertility and irregular menstrual cycles over two years. Physical examination was consistent with abdominal tenderness in the lower abdomen. During the genital examination, a tender mass was found and confirmed with pelvic ultrasonography and contrast MRI, documenting a homogeneous mass lesion right para uterine measuring 10.2×7.6 cm with high vascularization after IV contrast administration (Figure 1, panels A and B). Also, a cystic lesion measuring 5.6×4 cm was observed in the left ovary. Due to high vascularization observed during imaging studies, the possibility of malignancy was considered, and surgery with intraoperative biopsy was recommended. During surgery, the lesion was observed on the fundus and anterior region of the uterus. Tumor excision was performed and sent for analysis; another lesion with irregular borders and thickness of the myometrium was observed on the right lateral side, which was also excised and sent for extemporaneous biopsy (Figure 1, panel C).

Right adnexa was unremarkable. Left adnexa presented a pearly white tumor of 5×5 cm, and excision of the lesion was performed. Intraoperative histological study revealed high cellularity with the presence of sheets of cells arranged in clusters with hyperchromatic nuclei and coarse chromatin (Figure 1, panel D). Possibilities of atypical cellularity were suggested. Macroscopically, the tumor appeared localized in the myometrium, and no apparent lymph node abnormalities were documented. Due to the patient's age and fertility wishes, local excision was performed, preserving the uterus. The final pathology study reported the same findings, with cells presenting a good nucleus cytoplasm ratio. Groups of cells with swirling patterns, loose and vascularized stroma mixed with endometrial glandular groups of different sizes with surrounding stroma and hemosiderin within the muscular bundles were identified. A final diagnosis of highly cellular leiomyoma with a focus of adenomyosis was given based on overall histomorphology. The patient recuperated satisfactorily after surgery with no further interventions required.

## Discussion

Preservation of fertility is often preferred in premenopausal women, and conservative surgical treatment such as myomectomy for leiomyomas may be offered, which is the case of our patient who presented to the clinic with fertility wishes [1]. Our patient manifested irregular menstrual cycles and lower abdominal pain during examination associated with infertility. Women with leiomyomas and adenomyosis are more prone to develop stronger symptomatology [8].

Once the clinical examination as part of the gynecological examination does not reveal significant findings, it is recommended to complement the evaluation with paraclinical examinations, which include ultrasound and, in some cases MRI, which has been utilized as a helpful tool to diagnose uterine lesions [6,9].

Leiomyoma tissue is poorly vascularized, containing smooth muscle fiber bundles and extracellular matrix material (ECM) composed of collagen, fibronectin, laminin, and proteoglycans [2]. Although this patient’s MRI with IV contrast reported a para uterine tumor highly vascularized, it is noteworthy that perfusion images do not distinguish between atypical leiomyomas and sarcomas, since even benign leiomyomas could be highly vascular [1]. In contrast, color Doppler US helps distinguish focal adenomyosis from leiomyomas, which tend to displace vessels and show circumferential flow, the usual pattern observed in this case [10].

Based on clinical and image findings, our patient was offered surgical treatment. It is well-known that normal myometrial smooth muscle cells grow uniformly and parallel to the major axis of the cell, whereas uterine leiomyoma cells form globular aggregates or nodules, contributing to the swirled pattern [3]. Surgical findings in our patient consisted of one lesion with characteristics of fibroids. A second lesion was found deeper into the myometrium, denser, with irregular borders, and highly vascularized. The microscopic study reported a highly cellular leiomyoma with a focus of adenomyosis.

The case studies in Chapron et al. have shown smooth muscle growing as benign masses around endometrial tissue, usually exhibiting well-defined organization in the myometrium. Smooth muscle cells are increased in number and size compared to the surrounding myometrial cells [6]. Because the ectopic endometrial glands remain hormonally sensitive, they can provoke a local stimulation in the muscular tissue with subsequent hyperplasia and hypertrophy. Myometrial thickening can present different patterns, although focal areas have relatively indistinct borders compared to leiomyomas [10].

Cellular leiomyoma is a histological subtype with more compact smooth muscle cells and less collagen, showing in MRI a substantial enhancement after injection of contrast agent [9]. These features were observed in our patient.

In some situations, distinguishing between an ovarian lesion and a uterine mass is complex, and several signs have been described to differentiate the location of the ovarian lesion from the uterine mass [9]. That was the case of our patient, in which an ovarian lesion was identified by images and excised during surgery. The fact that the leiomyomas were located on the right side of the uterus made the visualization of the left ovary and subsequent benign tumor excision more approachable.

## Conclusions

Uterine fibroid is a benign condition found in different locations along the uterus and is associated with additional symptomatology that requires surgical intervention in many cases. The importance of adequate interpretation of images before surgery to identify possible features that can guide a malignant diagnosis of leiomyosarcoma or, in better circumstances, an associate condition like adenomyosis is noteworthy. Also, it is relevant to consider the presence of histological variations of leiomyoma, like high cellularity, that could make the surgical approach more difficult during the excision in patients with fertility wishes. Therefore, the relevance of performing extemporaneous biopsy to discard malignancy characteristic of the tissue with subsequent histology confirmation, especially in those cases like this one where high vascularity was observed before surgery and visible differences like thicknesses with irregular borders were seen. Although the presence of high cellularity and an adenomyosis focus are benign diagnoses that do not differ the conservative versus radical surgical treatment, histological confirmation is the clue for a successful outcome after surgery.
